# COVID-19 Assessment with Bedside Lung Ultrasound in a Population of Intensive Care Patients Treated with Mechanical Ventilation and ECMO

**DOI:** 10.3390/diagnostics10070447

**Published:** 2020-07-02

**Authors:** Hasse Møller-Sørensen, Jakob Gjedsted, Vibeke Lind Jørgensen, Kristoffer Lindskov Hansen

**Affiliations:** 1 Department of Cardiothoracic Anaesthesiology, Copenhagen University Hospital, Rigshospitalet, 2100 Copenhagen, Denmark; jakob.gjedsted@regionh.dk (J.G.); vibeke.lind.joergensen@regionh.dk (V.L.J.); 2 Department of Clinical Medicine, University of Copenhagen, 2100 Copenhagen, Denmark; Kristoffer.Lindskov.Hansen.01@regionh.dk; 3 Department of Radiology, Copenhagen University Hospital, Rigshospitalet, 2100 Copenhagen, Denmark

**Keywords:** COVID-19, ECMO, veno-venous ECMO, VV-ECMO, LUS, lung ultrasound, LUS score, lung ultrasound score

## Abstract

The COVID-19 pandemic has increased the need for an accessible, point-of-care and accurate imaging modality for pulmonary assessment. COVID-19 pneumonia is mainly monitored with chest X-ray, however, lung ultrasound (LUS) is an emerging tool for pulmonary evaluation. In this study, patients with verified COVID-19 disease hospitalized at the intensive care unit and treated with ventilator and extracorporal membrane oxygenation (ECMO) were evaluated with LUS for pulmonary changes. LUS findings were compared to C-reactive protein (CRP) and ventilator settings. Ten patients were included and scanned the day after initiation of ECMO and thereafter every second day until, if possible, weaned from ECMO. In total 38 scans adding up to 228 cineloops were recorded and analyzed off-line with the use of a constructed LUS score. The study indicated that patients with a trend of lower LUS scores over time were capable of being weaned from ECMO. LUS score was associated to CRP (*R* = 0.34; *p* < 0.03) and compliance (*R* = 0.60; *p* < 0.0001), with the strongest correlation to compliance. LUS may be used as a primary imaging modality for pulmonary assessment reducing the use of chest X-ray in COVID-19 patients treated with ventilator and ECMO.

## 1. Introduction

In December 2019, several cases of a severe pneumonia were reported in Wuhan, China. Analyses of the patients revealed that a novel coronavirus was the cause of the outbreak [1]. The novel coronavirus was named SARS-CoV-2 by the World Committee on Virus Classification, and the disease caused by the virus was named COVID-19 by the World Health Organization [2].

The coronavirus with a crown shaped viral envelope, has a high infectivity and can cause a variety of symptoms; headache, dry cough, dyspnea, myalgia, fatigue and fever are reported [1,2]. Imaging of the coronavirus pneumonia has mainly concerned computed tomography (CT) and chest X-ray [3,4,5]. Previous imaging studies show that patients with COVID-19 exhibit subpleural consolidations with ground glass opacities (GGO), interlobular septal thickening, crazy paving and pleural thickening.

Lung ultrasound (LUS) is an emerging imaging modality for lung assessment in intensive care units with the ability of bedside and real-time assessment of pulmonary function and changes [6,7,8]. Several studies have shown that LUS is useful for diagnosing acute pulmonary conditions, such as pulmonary edema and pneumonia [9,10,11,12]. As COVID-19 gives rise to peripheral pulmonary changes, LUS could be a potentially useful modality for disease monitoring in the intensive care unit. Furthermore, COVID-19 is a highly infectious disease with a marked risk of virus exposure to healthcare workers [13], which may be reduced by introducing bed-side LUS for treatment guidance as an alternative to chest X-ray and CT examinations. Few papers have investigated LUS in relation to COVID-19 disease showing that pulmonary changes may be visualized with ultrasound [14,15].

In this prospective study, patients with verified COVID-19 disease hospitalized at the intensive care unit and treated with mechanical ventilator and extracorporal membrane oxygenation (ECMO) were evaluated with LUS for pulmonary changes. LUS findings were compared to C-reactive protein (CRP) and ventilator settings. The aim of this study was to investigate in severely diseased COVID-19 patients hospitalized in an intensive care unit if LUS can assess the pulmonary changes, and if a LUS score can determine the progress of the disease.

## 2. Materials and Methods

A total of 10 patients (6 males) with a median age of 52.6 years (range: 28–65 years) with COVID-19 disease and hospitalized at the intensive care unit were included in the study. The study was approved by the regional data protection agency. The local ethics committee waived ethical approval, as ultrasound scanning of the lungs is considered a routine procedure (22th November 2016, no.: 16031001).

**Figure 1:**
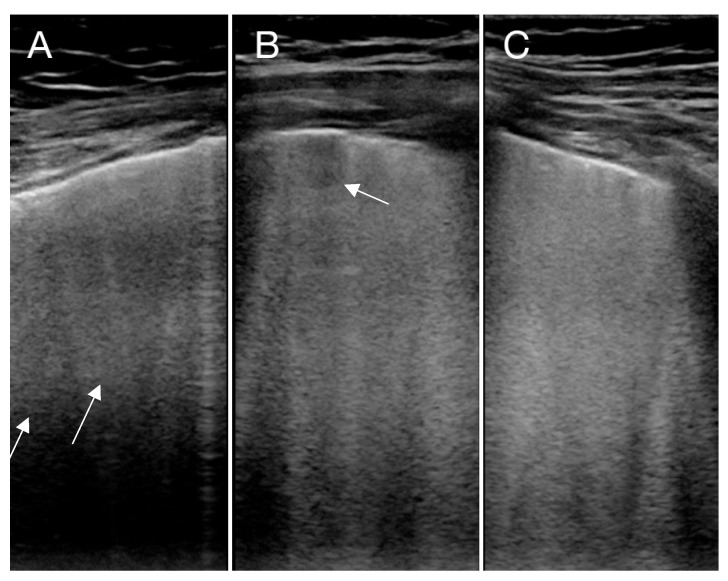
Lung ultrasound (LUS) of three different patients with COVID-19 showing multifocal (**A**,**B**) (marked with an arrow) and confluent B-lines (**C**). In (**B**), a small subpleural consolidation is also seen (marked with an arrow).

**Figure 2:**
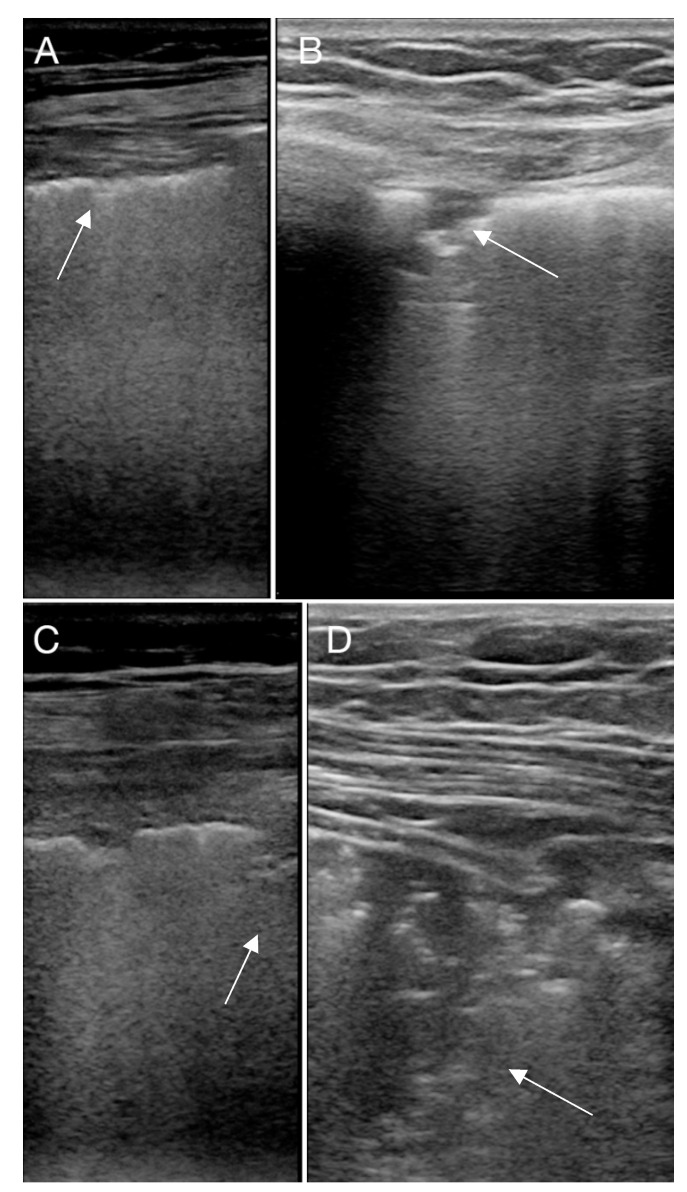
An irregular pleural line is observed in (**A**) (marked with an arrow). In (**B**,**C**) small subpleural consolidations are found (marked with an arrow), while in (**D**), a larger subpleural consolidation with air-bronchogram is found (marked with an arrow).

**Figure 3:**
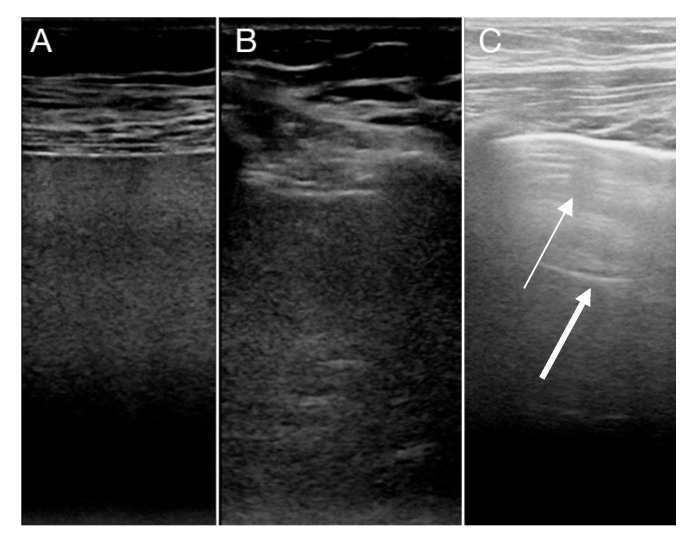
Large consolidations with hepatization in (**A**,**B**). Note the absence of A- and B-lines. In (**C**), LUS of a normal lung segment is shown with focal B-lines and A-lines present (thin arrow points to B-Lines and the bold arrow to A-line).

All patients were on a mechanical ventilator and treated with veno-venous-ECMO (VV-ECMO). They were examined with LUS on day 1, 3 and 5 in the intensive care and before weaning from VV-ECMO. LUS was performed with a Philips Cx 50 (Phillips Healthcare, Amsterdam, The Netherlands) using a 12 mHz linear probe (L12). Settings were set to penetration mode with a low center frequency and with a single focus zone at the pleural level. The depth was adjusted so that 5–6 cm of the lung tissue was visualized. No compound or harmonics was used. LUS was performed along the ribs with cineloops of 3 s. Each lung was divided in to 3 zones; anterior, lateral and posterior, as recommend by others [16,17]. For each zone, pleural and lung diagnostics were recorded according to Table 1 and assessed as recommended by other authors [14,15]. Examples of pulmonary changes seen with LUS in examined COVID-19 patients are given in Figure 1, Figure 2 and Figure 3.

To assess the lung changes, a scale was generated, where observations for each zone were added to a total score for each patient. A maximum of 17 points could be given in each zone, adding to a total maximum score of 102 for each patient. The scoring system is given in Table 1. Cineloops of the LUS examination were examined off-line and scored by one experienced intensivist blinded to other parameters. In addition to the LUS examination, the CRP and ventilator setting, i.e., dynamic compliance, were recorded for each patient, Additionally, LUS was compared to corresponding chest X-ray for a visual qualitative evaluation by an experienced radiologist. A chest X-ray was performed with a mobile X-ray unit (!M1, Solutions-For-Tomorrow, Vackelsang, Sweden).

### Statistics

Data were initially analyzed with descriptive statistics. Associations between LUS score, CRP and compliance were tested with Pearson’s correlation. Significance level was set to 0.05, and statistical analyses were performed with IBM SPSS Statistics v.19 (SPSS Inc., Chicago, IL, USA).

## 3. Results

**Figure 4:**
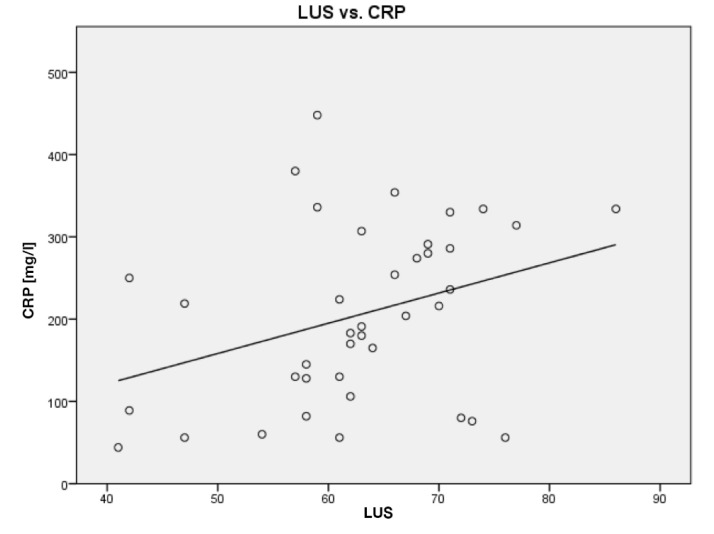
LUS score compared to C-reactive protein (CRP). Line of best fit is shown as a solid line.

**Figure 5:**
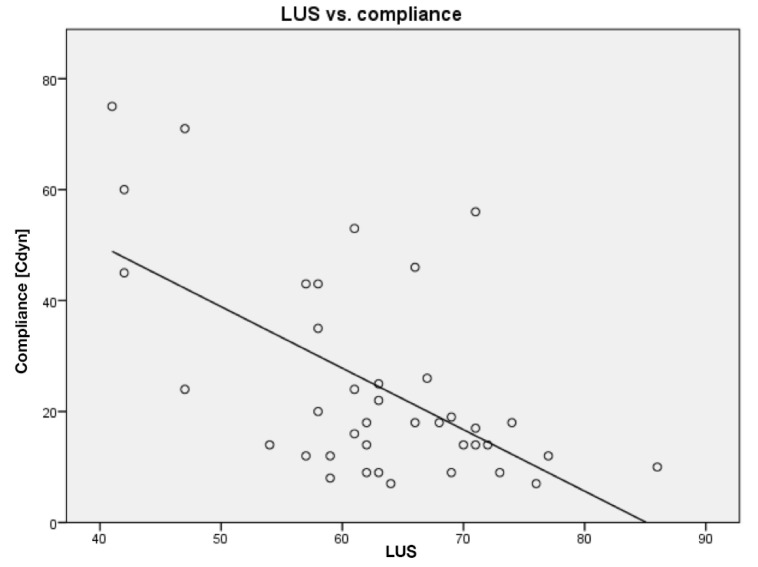
LUS score compared to compliance. Line of best fit is shown as a solid line.

Ten patients were included in the study, and all patients were scanned the day after initiation of VV-ECMO and thereafter every second day until, if possible, being weaned from VV-ECMO. In total, the patients were scanned 38 times adding up to 228 cineloops being analyzed off-line. An overview of data is given in Table 2. When comparing LUS score with CRP (*R* = 0.34; *p* < 0.03) and compliance (*R* = 0.60; *p* < 0.0001), correlation analyses show significant associations. Scatterplots are given in Figure 4 and Figure 5.

**Figure 6:**
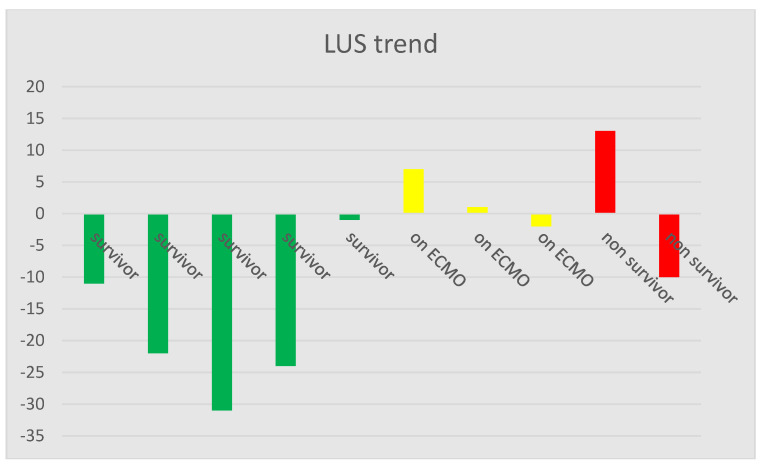
Box plot of the LUS trend calculated from the LUS scores of the first and last day of scan for each patient.

When comparing the LUS score over time, there was a trend suggesting that patients with a lower score over time were capable of being weaned from VV-ECMO, as shown in Table 3 and illustrated in Figure 6. Patients with no major changes in the LUS score were still on VV-ECMO and presented major changes in lung function when recording of data was ended. In the non-survivor group, patient 9 showed the largest increase in the LUS score and died on VV-ECMO, because of worsening of the condition with spontaneous bleeding into the pleural cavity. Patient 10 showed a decrease in LUS score and improvement in lung function, CRP and compliance but died from intraabdominal bleeding, and is therefore an outlier, as seen in Table 3 and Figure 6, with the impact on mean scores in Table 2.

**Figure 7:**
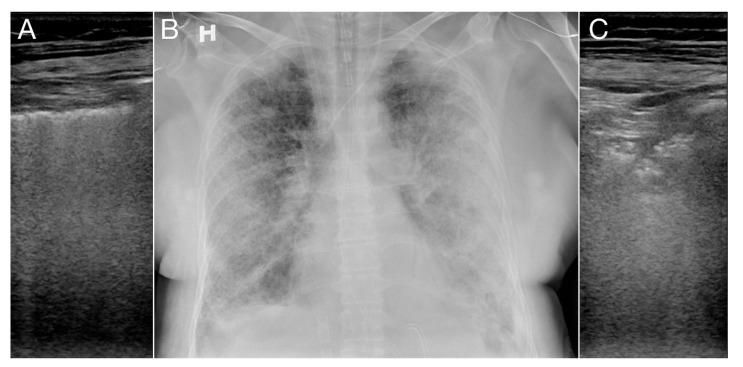
A patient with corresponding LUS and chest X-ray is shown. The ultrasound images are taken from the lateral view of each lung. The interstiel edema in the right lung visible with LUS as increased B-lines (**A**) is seen as numerous kerley B-lines in the chest X-ray (**B**). The consolidations with air-bronchograms in the left lung visible with LUS (**C**) are seen as subpleural consolidations in the chest X-ray (**B**).

It was observed in the group weaned from VV-ECMO that both the reappearance of lung sliding in more than two out of the six zones and regression of subpleural consolidations were signs of improvement of the lung function, indicating that the patients were ready to be weaned from VV-ECMO. For selected patients, corresponding chest X-ray and LUS were compared and revealed no unexpected findings. By visual inspection, the changes found with the two modalities were comparable, as illustrated in Figure 7.

## 4. Discussion

This study shows that LUS is a useful and a valuable tool for monitoring patients with severe COVID-19 disease. The patients showed peripheral changes in the lung tissue visible with LUS. Severe changes were consolidations and a thickened pleural line. B-lines were multifocal or confluent. When disappearing, it was a sign of deterioration of the condition with increasing consolidation depleting air from the subpleural lung tissue. With improvement, consolidations became less solid with air bubbles i.e., the appearance of air bronchograms.

Five patients had pleural effusion, and for two of these patients, the fluid appeared on day 2, while the others had fluid from the beginning of the ECMO treatment. It has been stated that COVID-19 is not associated with a high risk of pleural effusion, however, in the most diseased patients it apparently appears more frequently [18].

COVID-19 gives rise to thickened pleura, which has been observed by Peng et al. Along with pleural changes, the artefacts arising from the pleural line also change. B-lines are reverberations artefacts generated by multiple reflections of the US beam trapped in air- and water-rich structures of the pleural line [19,20]. Increasing B-lines are found with increasing interstitial lung water, and are used as an indicator of pulmonary edema. One of the early signs of COVID-19 are increasing B-lines [21]. All patients in this study had severe pulmonary changes with consolidations and pleural thickening. The B-lines assessment was useful, as affected lung zones had multiple B-line artefacts, as seen in Figure 1.

A-lines are reverberation artefacts arising between the pleural line and the transducer surface [22]. In the patients of this study, few A-lines were visible, as the thickened irregular pleural surface cancelled the reverberation. Hence, A-line diagnostic was an easy measure for pleural assessment. Peng et al. has stated that the reappearance of A-lines is a sign of recovery, and this was also seen for some of the patients in this study [16].

With larger consolidations, A- and B-lines disappeared, and the lung tissue appeared more homogenous with reduced lung sliding. In overweight patients, large consolidation can be difficult to assess with LUS. An aid for diagnosing larger consolidations in LUS was inspection of the pleural line, as it became thinner and less bright, when the lung tissue below was consolidated (Figure 3). Additionally, vessels in the consolidated lung tissue were visible, resembling liver tissue, and therefore, large lung consolidations in LUS are named hepatization of the lung. Hence, the disappearance of A- and B-lines combined with less prominent pleural line and reduced lung sliding are worrisome features, indicating large consolidations.

In this study, a scoring tool for lung assessment using LUS was introduced, similar to the scoring systems proposed for LUS of adult patients in intensive care [23,24]. LUS can be limited by observer dependency. With introduction of a scoring system for LUS, a more reliable and observer independent tool for decision-making in the treatment of COVID-19 may be achieved. In this preliminary study, associations between LUS score, compliance and CRP were significant, and a decreasing trend in LUS score indicated a treatment response. LUS score had a stronger correlation to compliance than to CRP. While dynamic compliance is a marker of respiratory function and based on chest wall compliance, lung tissue compliance and airway resistance, CRP is an acute phase reactant and an unspecific biomarker of systemic inflammation, which may explain the different correlations found.

Several limitations in this preliminary study should be addressed. The study was limited by a small patient population, and no inter- or intraobserver variation for LUS score was assessed. Future studies with more patients are warranted, to validate the LUS score and to identify cutoff levels for weaning off VV-ECMO and futile treatment. Patient overweight was a limitation in the LUS examination, reducing penetration of the ultrasound to the pleura and the subpleural areas. As all patients were supine in bed, a thorough examination of the lungs was difficult to perform for some patients. In this study, a linear array was used, and it could have added to the value of the study, if a curved array or micro-convex array had been used for larger patients as done in other studies [11,25]. The scanner did not have a dedicated lung setup, and a vascular setup with low center frequency was used instead. As LUS in many intensive care departments is rarely performed, the present scan situation with US equipment not intended for LUS might be a common limitation. Nevertheless, LUS was feasible in all patients, and valuable information was extracted. In this study, only six zones were investigated. Other groups have recommended up to 28 zones for LUS examination [12,20,26]. However, with a six zone LUS, an overall impression of the lung status was achieved. The LUS examination took around 5 min, which is acceptable in a busy work environment. Patients on VV-ECMO are exposed to multiple interventions that may influence pulmonary function and biomarkers, which may have affected the results. None of the patients included in this study received anti-inflammatory drugs.

Bedside LUS in the intensive care unit is a valuable, accessible and reliable tool for COVID-19 assessment to the intensivist. Bedside LUS can be used as a primary imaging modality and reduce the need for a chest X-ray. LUS may reduce ionizing of the COVID-19 patient, as well as workload and infections risk for the radiographers. We therefore encourage intensivists to take LUS into the COVID-19 departments for lung assessment.

## 5. Conclusions

Patients with COVID-19 in intensive care treated with VV-ECMO can be monitored with LUS, providing valuable information on pulmonary function over time. Using a score for pulmonary changes, LUS may be used to distinguish patients with improving pulmonary function from patients with stationary or deteriorating pulmonary function. The LUS score showed a moderate to strong correlation with compliance, though larger studies that include more patients are needed to confirm the results. This study indicates that LUS may be used as a primary imaging modality for COVID-19 assessment, reducing the use of chest X-ray.

## Figures and Tables

**Table 1:** Table for LUS findings according to lung zones, along with a constructed scoring system for observed lung changes.

	Table for LUS Findings of Left/Right Lung			Scoring System for LUS Findings		
	Anterior	Lateral	Posterior			
Pleural thickening (Y/N)				Yes +3		No 0
Lung sliding (Y/N)				No +3		Yes 0
B-line appearance (none/multifocal/few)				None +3	Multifocal +2	Few +1
A-line appearance (Y/N)				No +3		Yes 0
Subpleural consolidation (Y/N)				Yes +2		No 0
Lobar consolidation (Y/N)				Yes +2		No 0
Pleural fluid (Y/N)				Yes +1		No 0

**Table 2:** Overview of data. “Survivor” defines patients being weaned from veno-venous-extracorporal membrane oxygenation (VV-ECMO), “On ECMO” defines patients still on ECMO at study end, and “Non-survivor” defines patients who died on ECMO. Standard deviations are given in parenthesis.

	Total (*n* = 10)	Survivor (*n* = 5)	On ECMO (*n* = 3)	Non-Survivor (*n* = 2)
LUS score	62.7 (9.9)	60.4 (9.1)	68.8 (8.0)	61.2 (11.5)
CRP [mg/L]	205.1 (107.6)	189.5 (116.6)	238.8 (81.4)	202.3 (114.7)
Compliance [Cdyn]	24.7 (18.4)	25.7 (17.0)	14.8 (5.2)	33.9 (26.0)

**Table 3:** Findings in 10 patients included. Start and end define first and last day of scan. End for “Survivor” group is before weaning from VV-ECMO, for “On ECMO” group at last day of study and for “Non-survivor” group before death.

Patient No.	Patient Status	LUS Start	LUS End	CRP Start [mg/L]	CRP End [mg/L]	Compliance Start [Cdyn]	Compliance End [Cdyn]
1	survivor	74	63	334	191	18	25
2	survivor	69	47	291	56	19	24
3	survivor	72	41	80	44	14	75
4	survivor	66	42	354	89	46	45
5	survivor	59	58	448	128	12	35
6	on ECMO	63	70	180	216	22	14
7	on ECMO	61	62	130	106	16	9
8	on ECMO	71	69	330	280	14	9
9	non-survivor	63	76	307	56	9	7
10	non-survivor	57	47	380	219	43	46
